# Molecular markers for the efficacy of neoadjuvant immunotherapy for head and neck squamous cell carcinoma

**DOI:** 10.3389/fonc.2025.1586130

**Published:** 2025-06-23

**Authors:** Jiabin Zhu, Yudong Ning

**Affiliations:** ^1^ Department of Neurology, Teng Zhou Central People’s Hospital, Tengzhou, China; ^2^ Department of Head and Neck Surgery, National Cancer Center/National Clinical Research Center for Cancer/Cancer Hospital, Chinese Academy of Medical Sciences and Peking Union Medical College, Beijing, China

**Keywords:** head neck squamous cell carcinoma, immunotherapy, molecular marker, tissue biopsy, liquid biopsy, radiomics, artificial intelligence

## Abstract

Cancer ranks among the most formidable diseases. Currently, the treatment of malignant tumors has entered the immunotherapy era. Immunotherapy has achieved remarkable progress in treating malignant tumors, including head neck squamous cell carcinoma. Nevertheless, a significant number of patients exhibit a limited response to this treatment. Thus, the quest for novel molecular biomarkers to assess the efficacy of immunotherapy is of utmost importance. In recent years, the prediction and evaluation of immune efficacy have emerged as focal points of research. Biomarkers developed based on tissue biopsies (such as programmed death ligand-1 expression, tumor infiltrates lymphocyte subsets, tumor mutation burden, cancer-associated fibroblasts, etc.), liquid biopsies (circulating tumor DNA, circulating tumor cells, and extracellular vesicles, etc.), when combined with nanotechnology, have shown the potential for highly sensitive prediction. This is achieved through non-invasive real-time monitoring of clonal evolution and immune escape. Moreover, radiomics and artificial intelligence (such as deep-learning models) can noninvasively predict and evaluate treatment response and prognosis. In this study, we comprehensively summarize the research progress of molecular markers for predicting and evaluating the efficacy of immunotherapy in head neck squamous cell carcinoma. We approach this from the perspectives of tissue biopsy, liquid biopsy, radiomics, and artificial intelligence.

## 1 Introduction

**Figure 1:**
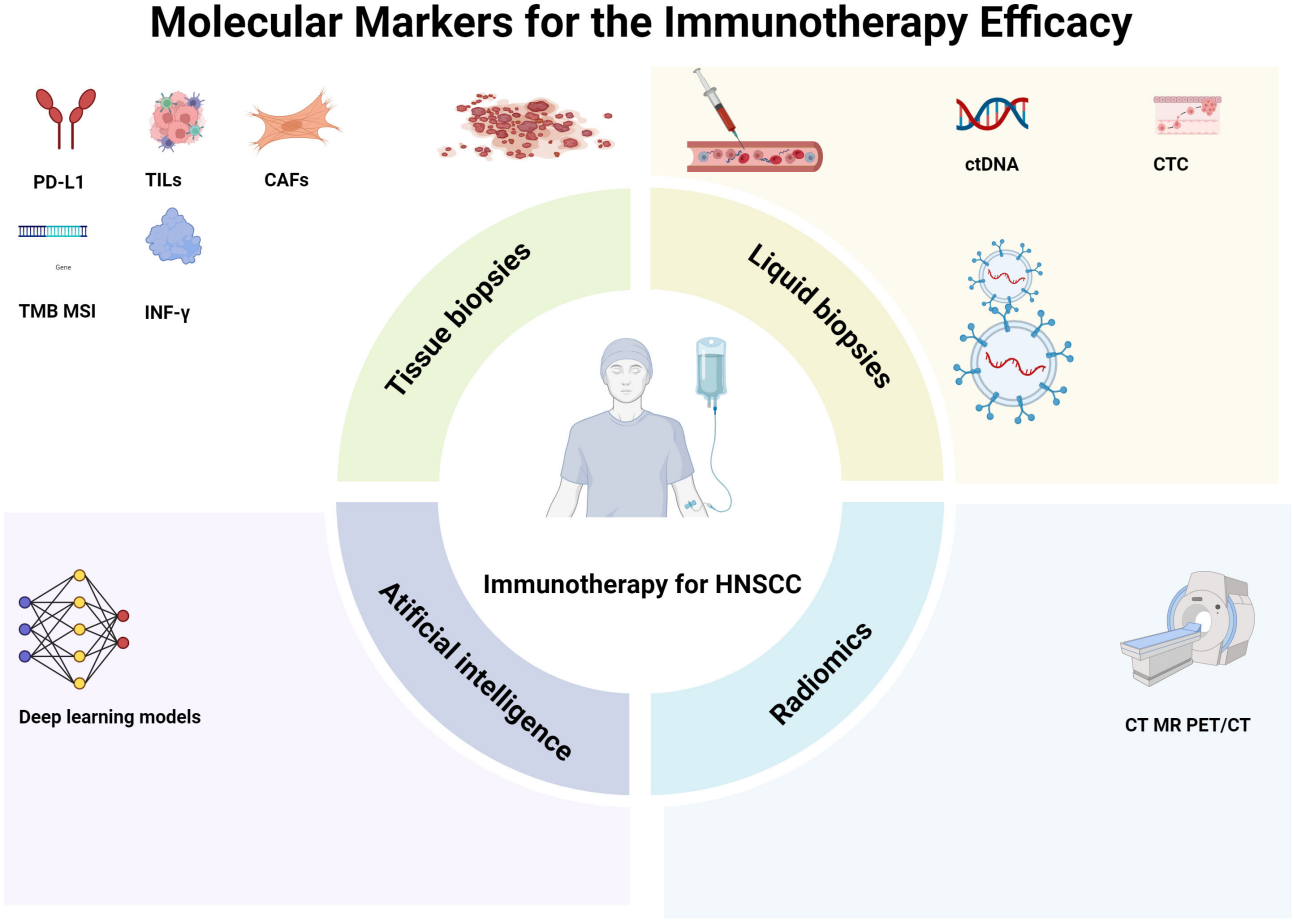
Molecular markers for the immunotherapy efficacy. Biomarkers developed based on tissue biopsies, such as programmed death ligand-1 (PD-L1) expression, tumor cell subpopulations, tumor mutation load, and fibroblasts, are important. In addition, liquid biopsies, including circulating tumor DNA (ctDNA), circulating tumor cells (CTCs), and extracellular vesicles (EVs), also play significant roles. Moreover, radiomics, which involves computed tomography (CT), magnetic resonance imaging (MRI), and positron emission tomography/computed tomography (PET/CT), and artificial intelligence, such as deep-learning models, can non-invasively predict and evaluate treatment response and prognosis. HNSCC head neck squamous cell carcinoma.

Head neck squamous cell carcinoma (HNSCC) is a prevalent malignant tumor. Surgery and radiotherapy serve as the primary treatment modalities for HNSCC patients. Regrettably, the vast majority of patients present at the middle or late stages of the disease. Even after undergoing a series of comprehensive treatments including surgery, radiotherapy, and chemotherapy, the recurrence and mortality rates remain distressingly high. Immunotherapy has emerged as a rapidly-developing treatment approach in recent years. Tumors evade the immune system by expressing immune checkpoint ligands, such as Programmed death receptor-1 (PD-1), Programmed death ligand-1 (PD-L1), cytotoxic T-lymphocyte-associated protein 4, and others (1). Based on clinical trials like Keynotes-012, KEYNOTE-040, KEYNOTE-048, and CheckMate141, immune checkpoint inhibitors (ICIs) have been shown to extend the overall survival of patients with recurrent/metastatic HNSCC (2–5). Nivolumab and pembrolizumab were approved by the U.S. Food and Drug Administration (FDA) in 2016 for use as first-line treatments for recurrent/metastatic HNSCC. Inspired by these promising results, numerous centers have initiated explorations into neoadjuvant immunotherapy for locally advanced HNSCC and have achieved satisfactory outcomes (6–8). Neoadjuvant ICIs combined with chemotherapy represent a commonly-employed treatment regimen (9). Nevertheless, clinical observations reveal that not all patients respond favorably. Some patients show poor responses, and current screening methods are still insufficient. There is thus an urgent need for reliable predictive biomarkers to facilitate personalized clinical management and the development of new treatment strategies. Regarding the screening of biomarkers for immunotherapy, tissue biopsy and liquid biopsy are the two principal methods for sample collection in clinical practice (10). Tissue biopsy mainly involves analyzing factors such as the expression of PD-L1 in tumor tissues, tumor lymphocyte infiltration, tumor mutational burden (TMB), tumor infiltrates lymphocyte subsets (TILs) and cancer-associated fibroblasts (CAFs) within tumor tissues. However, tissue biopsy is plagued by issues of spatial and temporal heterogeneity. Liquid biopsy, on the other hand, is carried out by collecting peripheral blood to detect circulating tumor DNA (ctDNA), circulating tumor cells (CTC), and extracellular vesicles (EVs). It is a highly efficient and non-invasive method for dynamically assessing the efficacy of immunotherapy. The development of nanomaterials, nanostructures, and nanotechnology has significantly enhanced the detection efficiency of liquid biopsy, which is currently a hot topic in clinical research. Advances in imaging and the ascent of artificial intelligence are also being harnessed to screen for predictive biomarkers of immunotherapy. In this review, we will summarize the current biomarkers for predicting and evaluating the efficacy of immunotherapy in HNSCC from the perspectives of tissue biopsy, liquid biopsy, imaging examination, and artificial intelligence, with the aim of better guiding clinical practice (**Figure 1**, **Table 1**).

**Table 1:** Comparison of key molecular markers for immunotherapy efficacy.

Types	Molecular markers	Mechanism	Advantages	Disadvantages
**Tissue biopsies**			Gold standard;Providing richer pathological features	Temporal and spatial heterogeneity
	PD-L1	Suppressed T cell; Escaped immune killing	Gold standard	The prediction efficiency is not enough, and supplemented biomarkers are needed
	TILs	Tumor cytolysis and maintained immune surveillance	Predictive model of TILs and PD-L1	Need verification in untreated HNSCC
	CAFs	Immunosuppression and stimulating Tregs and cytotoxic T cells	Immunosuppressive HNCAF-1 specific in HNSCC	Need verification in larger number of untreated HNSCC
Liquid biopsies			Non-invasive and reflecting the overall state of the human body	Low sensitivity and specificity, limited clinical application
	ctDNA	Multi-faceted analysis of tumor genes	Rich information	The numbers are small, require a large number of blood samples, and the mutations identified may also reflect non-malignant cells, leading to false-positive results
	CTC	Represent the heterogeneity of parental cells	CTCs, as a means of liquid biopsy for detecting PD-L1 expression represents a highly viable technical approach	Short life, small quantity, low concentration, dynamic heterogeneity and so on
	EVs	Represent the heterogeneity of parental cells	EVs contains a variety of molecular makers and is stable and easy to preserve	Limited clinical application
**Radiomics**	Extracted parameter features	The immunotherapy efficacy was predicted by imagine features	Non-invasive	Limited clinical application
**Artificial intelligence**	Data analysis	Multi-omics datasets in oncology	Non-invasive, interdisciplinary	Limited clinical application

Bold means diagnosis methods.

PD-L1 Programmed death ligand 1; TILs Tumor infiltrates lymphocyte subsets; CAFs cancer-associated fibroblasts; ctDNA circulating tumor DNA; CTC circulating tumor cell; EVs Extracellular vesicles; HNSCC head neck squamous carcinoma.

## 2 Molecular markers commonly used in tissue biopsy

Cytological puncture or tissue biopsy is a frequently employed method for diagnosing malignant tumors. Moreover, numerous biomarkers relevant to immunotherapy are also evaluated through tissue biopsy procedures.

### 2.1 PD-L1

Ever since ICIs came into use for the treatment of HNSCC, PD-1 and PD - L1) have attracted substantial attention. In clinical practice, the expression level of PD-L1 is typically detected via immunohistochemistry of tumor biopsy tissue. This detection serves the purpose of screening patients who are likely to benefit from ICIs (3, 11, 12). PD-1 is expressed by T cells following their activation and binds to one of its ligands, namely PD-L1 or Programmed Death Ligand–2(PD-L2). The interaction between PD-L1 and its receptor PD-1 leads to a reduction in the activation and proliferation of T cells. Consequently, this enables tumors to evade the immune response. PD-L1 is expressed on a diverse range of cells, encompassing tumor cells as well as immune cells such as activated lymphocytes, macrophages, dendritic cells, and mast cells (13–15). The Tumor ratio score (TPS) and the combined positive score (CPS) are two important immune indexes utilized for evaluating the efficacy of ICIs. TPS is defined as the expression of PD-L1, calculated as the number of positive tumor cells divided by the total number of viable tumor cells, and then multiplied by 100%. In contrast, CPS is defined as the proportion of tumor cells (either partially or fully stained), lymphocytes, and macrophages that exhibit positive PD-L1 expression, relative to all tumor cells, multiplied by 100% (16). Although, theoretically, this value could exceed 100, the maximum CPS is conventionally defined as 100.In clinical trials, both of these scoring systems have employed various cutoff values. Currently, the consensus recommendation is to define PD-L1 positivity as TPS ≥ 1% or CPS ≥ 1 through immunohistochemical staining (17). The CPS holds a particularly superior value as a biomarker, especially when considering lower cutoff values (CPS ≥ 1) (18). When it comes to the role of PD-L1 testing in HNSCC, several crucial issues need to be taken into account (19). The first significant aspect pertains to the reproducibility of the staining protocol utilized for the immunohistochemical assessment of PD-L1 expression. The expression levels and staining distributions can vary substantially depending on the different protocols in use. This variability limits the ability to compare data obtained from different research centers. On the other hand, different observers may arrive at different interpretations of the results. Several studies have been conducted to evaluate the consistency of different staining protocols in assessing PD-L1 expression in HNSCC. These studies have also examined the inter-observer variability in evaluating the outcomes (20–23). Overall, there was moderate to significant agreement between the different PD-L1 assays, as well as inconsistencies between observers, especially when the assessment was performed by a trained pathologist.

### 2.2 TILs

The tumor immune microenvironment of HNSCC has been extensively investigated in multiple studies, which has led to the identification of immune checkpoints that play a role in suppressing the immune response. In this regard, TILs are regarded as a crucial effector of the anti-tumor immune response of the host. In the case of HNSCC, numerous studies have demonstrated that patients with elevated levels of CD3+ and CD8+ T cells tend to have a better survival outcome (24–28). In certain studies focusing on oropharyngeal cancer, the FoxP3+ regulatory T cells (Tregs) subgroup has been associated with a more favorable prognosis (29–32). Combining the assessment of TILs with the evaluation of PD-L1 expression represents an intriguing and promising approach. In fact, within HNSCC, there exists a positive correlation between the expression of PD-L1 and the presence of TILs (33, 34). Four distinct types of tumor microenvironments have been classified based on the presence or absence of TILs and PD-L1 expression. This classification aims to identify a more suitable framework for formulating immunotherapy strategies tailored to different tumor microenvironments (35). According to this classification system, they are categorized as follows: Type I features positive PD-L1 expression and a high density of TILs (PD-L1+ TIL+); Type II shows negative PD-L1 expression and a low density of TILs (PD-L1−TILs−); Type III is characterized by positive PD-L1 expression but negative TILs (PD-L1+ TILs−); and Type IV has negative PD-L1 expression yet positive TILs (PD-L1−,TILs+). Balermpas et al. (33) discovered that among 161 HNSCC cases, the group with a high ratio of CD8+ cells to PD-L1 expression had a significantly better prognosis compared to the group with a high CD8+ cell count but low PD-L1 expression. Additionally, they found that patients with tumors exhibiting a high CD8+/PD-L1 ratio were more prevalent in human papillomavirus (HPV)-positive tumors when compared to HPV-negative tumors.

Furthermore, HPV-positive HNSCCs are marked by a high expression of cytotoxic T lymphocyte antigen 4 (CTLA-4), as well as an increased presence of Tregs and PD-1+ lymphocytes (36–38). Nevertheless, although there is accumulating evidence indicating that the HPV status exerts a significant influence on the immune microenvironment of HNSCC, which might potentially alter the outcomes of biomarker evaluations used to predict the response to ICIs, there is currently no conclusive evidence establishing a relationship between the HPV status and immunotherapy, especially with regard to PD-1/PD-L1 inhibitors (24).

### 2.3 Abnormal expression of genes

TMB has emerged as a significant predictive biomarker for immunotherapy across several tumor types (39–41). TMB is defined as the total number of non-synonymous mutations present in each coding region of the tumor genome. It can be estimated through the use of next-generation sequencing-based techniques, such as gene-targeting panels. In the KEYNOTE study focusing on HNSCC (42), TMB was found to be capable of predicting the therapeutic response to pembrolizumab. Moreover, higher levels of TMB were associated with a longer progression-free survival. Analogous results were obtained in another study, where ICIs were shown to improve the overall survival of patients with higher TMB levels (43). Despite these promising findings, the absence of a standardized method for TMB assessment and reporting has, thus far, impeded its widespread clinical application in the context of HNSCC (44).

Another participation mechanism that increased TMB is associated with increased ICIs response is microsatellite instability (MSI) caused by DNA repair defects. However, given the very low frequency of MSI in HNSCC (1% to 3%), MSI testing is not routinely recommended (17).

Genomic analyses that are based on the assessment of immune-related gene expression or characteristics have been explored in a diverse range of solid tumors undergoing immunotherapy. These analyses generally demonstrate good predictive value for treatment response (44). Interferon-gamma (IFN-γ) is the sole member of Class II interferons and serves as a core molecule in the upstream pathway of PD-L1, stimulating its expression (45). IFN-γhas been shown to be significantly associated with the use of PD-L1 suppressive therapy and immune TME. IFN-γ plays a key driver role in predicting clinical response to treatment with PD-1/PD-L1 inhibitors (46). Current studies on HNSCC immunotherapy have revealed that differences in IFN-γ related genes are the predominant changes (47). In the KEYNOTE 012 study, researchers investigated the expression characteristics of IFN-γ-inflammatory immune genes in HNSCC patients treated with pembrolizumab. The results indicated that the expression values in responders were significantly higher than those in non-responders (48). The IFN-γ-related CXC chemokine family is involved in various tumorigenic processes, including tumor angiogenesis, immune cell infiltration, and leukocyte migration. It holds potential value in predicting and evaluating the efficacy of immunotherapy (2). Pan-cancer studies have demonstrated that CXCL9, as a predictor of ICIs, outperforms CD8 effector and T cell inflammatory signatures (47).

### 2.4 CAFs

CAFs are among the most crucial cell types within the tumor microenvironment of numerous cancers. Due to continuous activation, they are unable to undergo apoptosis (48). In recent times, studies have uncovered a relationship between CAFs and ICIs (49–51). When it comes to HNSCC, one particular study identified 14 gene expression clusters. This was achieved based on baseline samples and samples from patients treated with nivolumab. In this context, four subtypes of CAFs were categorized, namely HNCAF-0 to HNCAF-4 (52). Following immunotherapy, it was observed that the proportions of HNCAF-0 and HNCF-3 increased, whereas the levels of HNCAF-1 and HNCAF-2 decreased. Furthermore, CAFs possess the ability to secrete exosomes into cancer cells. These exosomes contain various components, including multi-drug resistance associated proteins, microRNAs (miRNA), and long non-coding RNAs (lncRNA) (53).

## 3 Temporal and spatial heterogeneity in tissue biopsy

### 3.1 Temporal heterogeneity

Nevertheless, tissue biopsy is confronted with the issue of temporal and spatial heterogeneity. In one study that delved into the alterations of CPS in primary tumors and in incurable local recurrent diseases subsequent to definitive curative treatments such as surgery, radiation therapy, or chemoradiotherapy, when the same cutoff value of CPS ≥ 1 was applied, significant discrepancies were detected in 36% of the cases (54). When higher cutoff values of ≥ 20 and ≥ 50 were utilized to evaluate the changes in CPS, studies consistently reported inconsistencies at rates of 32 - 33% (for the cutoff value of ≥ 20) and 20% (for the cutoff value of ≥ 50) following the intervention (55).This indicates that the immune status of patients changes dynamically during the course of treatment. However, repeated biopsies will inevitably cause damage to the patient. These findings strongly suggest that the immune status of patients undergoes dynamic changes throughout the treatment process.

### 3.2 Spatial heterogeneity

In HNSCC, it is quite common to encounter cases with early regional cervical lymph node involvement or even cases where the primary site of cervical lymph node involvement remains unknown. As a result, tissue from tumor-involved lymph nodes is frequently employed for histological evaluation. Given this situation, it is of great significance to understand the potential disparities in PD-L1 expression between primary tumors and synchronous lymph node metastases. This knowledge can potentially guide the selection of tissue samples for assessing PD-L1 expression. In fact, when Ambrosini et al. carried out a comparison between primary tumors and synchronous lymph node metastases, they discovered that when using cutoff values of CPS ≥ 1 and ≥ 20, the consistency rates were 93.3% and 80%, respectively (56). A comparable study evaluated the CPS in 38 cases of primary p16+ oropharyngeal squamous cell carcinoma and their matched synchronous lymph node metastases. The study found that all the investigated lesions had a CPS ≥ 1. However, when classifying the lesions as either low or high positive for PD-L1 based on a CPS cutoff value of ≥ 20, in 24% of the cases, the PD-L1 expression in the primary tumor did not match that in the lymph node metastases (57). It is worth noting that there is currently no clear evidence indicating whether PD-L1 is expressed at a higher level in primary tumors or in lymph node metastases. When comparing the primary tumor with distant metastases, the consistency of the CPS with the primary tumor was higher when the cutoff value was ≥ 1 (88.9%) compared to when the cutoff value was ≥ 20 (77.8%).

Moreover, due to the inherent randomness of the biopsy process, the sampled tissue cannot fully represent the entire tumor sample. This further exacerbates the issue of spatial heterogeneity, making it more challenging to accurately assess PD-L1 expression and draw reliable conclusions regarding the immune status of the tumor. This emphasizes the need for more comprehensive and representative methods for evaluating PD-L1 expression in HNSCC patients.

## 4 Molecular markers commonly used in liquid biopsy

Liquid biopsies involve the collection of biological fluids such as cerebrospinal fluid, saliva, pleural fluid, blood, ascites, and urine from cancer patients to diagnose cancer. Thanks to its flexible and non-invasive nature, liquid biopsy enables the dynamic monitoring of cancer recurrence, treatment efficacy, or the development of drug resistance. Tumors are characterized by strong heterogeneity. The genetic information of tumors can vary significantly between different individuals, within tumors located in different parts of the same individual, among different subclonal tissues in the same tumor location, and even among different cells within the same subclone. Traditional tissue biopsy, which involves taking a sample of diseased tissue for testing, has certain limitations. In contrast, liquid biopsies are theoretically more comprehensive and have a lower bias due to heterogeneity. Combining tissue biopsy with liquid biopsy has the potential to further increase the positive detection rate, thereby benefiting more patients.

Currently, liquid biopsy based on blood is the most crucial research area. It mainly focuses on detecting ctDNA, CTCs, and EVs in the blood. During the course of immunotherapy, liquid biopsy technology is employed to analyze and assess the microscopic changes that occur within the patient’s body after receiving treatment. This is highly beneficial for evaluating whether the tumor has progressed or not. By detecting the presence and changes of these molecular markers in the blood, clinicians can obtain valuable information about the tumor’s response to immunotherapy in a more timely and less invasive manner, which can guide subsequent treatment decisions and improve patient outcomes (58).

### 4.1 ctDNA

ctDNA primarily serves to detect the DNA fragments that are generated following the apoptosis, necrosis, and rupture of tumor cells. The identification of these DNA fragments within the bloodstream is highly advantageous for the early detection of tumors. Moreover, it enables a multi-faceted analysis of tumor genes, which in turn provides valuable guidance for subsequent treatment strategies (59). Specifically, after a patient undergoes three or more courses of anti-PD-1 antibody treatment, a decrease in the ctDNA level indicates that the immunotherapy is effective, and such patients tend to have a longer survival period. Notably, patients in whom ctDNA is undetectable after immunotherapy derive the greatest benefit and exhibit the longest survival duration. Additionally, research has revealed that changes in ctDNA can predict a patient’s response to immunotherapy several months earlier compared to radiological examinations. However, it should be noted that ctDNA constitutes only 0.1-10% of the total circulating free cell DNA (cfDNA) (60). Consequently, analyzing ctDNA typically necessitates a relatively larger blood sample volume. Furthermore, the mutations identified through ctDNA analysis may also originate from non-malignant cells, potentially leading to false-positive results (61). This highlights the need for further refinement of ctDNA detection techniques to improve their accuracy and reliability, while also emphasizing the importance of integrating ctDNA analysis with other diagnostic methods to enhance the overall assessment of tumor status and treatment response in the context of immunotherapy for HNSCC and other cancers.

### 4.2 CTCs

PD-L1 is a cell surface protein. The conventional approach for assessing PD-L1 involves immunohistochemical staining of focal tissue sections to ascertain whether there is abnormal PD-L1 expression on the surface of cancer cells. This traditional method has paved the way for the development of numerous diverse solutions for detecting PD-L1 through liquid biopsy. Firstly, the detection method that most closely resembles the traditional tissue section analysis is the immunohistochemical staining of CTCs. Currently, several studies have been carried out regarding the expression of PD-L1 in CTCs. The results clearly demonstrate a robust correlation between the PD-L1 expression in CTCs and that in tissue sections (62). Notably, in certain instances, the biological expression level of PD-L1 in CTCs is even higher than that in tissue sections. In HNSCC, CTCs can serve a dual role. They can function as a prognostic indicator, offering valuable insights into the patient’s disease prognosis. Additionally, they can act as a foundation for analyzing the molecular expression of biomarkers associated with immunotherapy response, such as PD-L1 and others (63). Hence, employing CTCs as a means of liquid biopsy for detecting PD-L1 expression represents a highly viable technical approach (64). Nevertheless, CTCs are characterized by a short lifespan, low abundance and concentration, and dynamic heterogeneity. Their isolation often relies on epithelial markers, and advanced technologies like microfluidic devices and enrichment strategies are required to enhance the sensitivity of detection (61, 65, 66).

### 4.3 EVs

**Figure 2:**
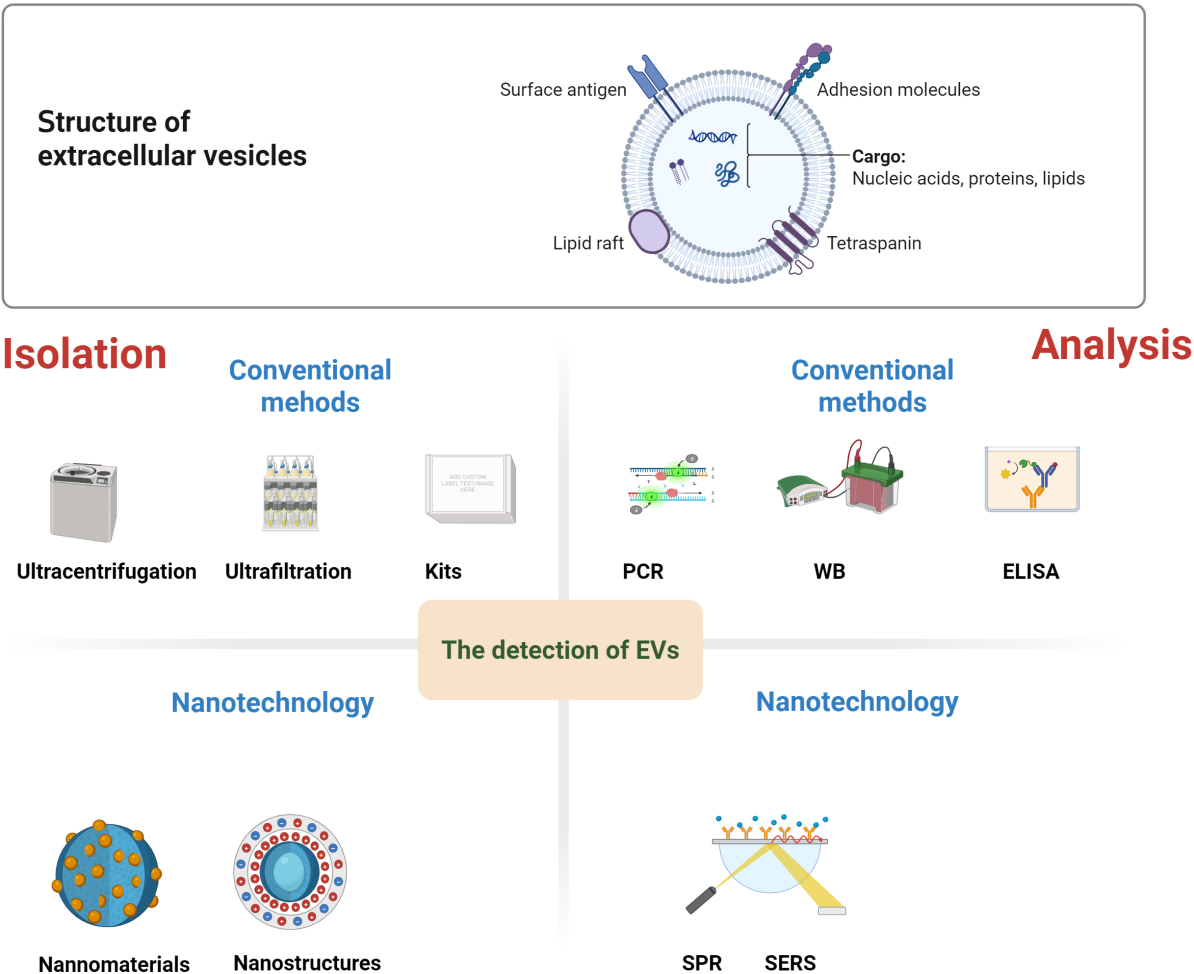
Structure and detection methods of EVs. EVs contain a plethora of biomolecules, including nucleic acid, proteins, metabolites, and lipids. There are conventional methods for EVs isolation, such as ultracentrifugation, ultrafiltration, or the use of kits. Similarly, conventional EVs analysis techniques includes polymerase chain reaction (PCR), Western blotting (WB), and enzyme-linked immunosorbent assay (ELISA). Nanomaterials, nanostructure have demonstrated remarkable advantages in the high-purity separation and high-sensitivity, high-specificity detection of EVs. Subsequently, the isolated EVs can be analyzed by using nanotechnology, including fluorescence, surface plasmon resonance (SPR), surface-enhanced Raman spectroscopy (SERS), etc.

EVs have emerged as a novel biomarker in liquid biopsy. EVs isolated from biological fluids consist of a diverse array of vesicles and nanoparticles, varying in cell origin, size, and concentration (67). Compared to ctDNA and CTC, EVs possess distinct advantages as follows (68, 69): 1. They are more abundant in biological fluids than CTC and carry more information than ctDNA. 2. EVs can be sourced from a wide range of biofluids, including blood, cerebrospinal fluid (CSF), urine, etc., while CTC and ctDNA are typically obtained from blood samples only. 3. EVs can traverse multiple cell membrane barriers, particularly the blood-brain barrier, which is of utmost significance for diseases in the central nervous system. 4. Due to their lipid bilayer structure, EVs are relatively stable and can be stored at - 80°C for an extended period while maintaining their morphology and content. Research has indicated that EVs play a role in mediating various biological pathways and mechanisms in cancer progression, such as cell growth, proliferation, and migration, by transferring EVs-encapsulated molecules between different cells. Thus, cancer- related molecules present in EVs are potential biomarkers for the diagnosis and prognosis of cancer patients (70). Shown in **Figure 2**, EVs contain a plethora of biomolecules, including DNA, messenger RNA (mRNA), microRNA (miRNA), long non-coding RNA (lncRNA), proteins, metabolites, and lipids, reflecting the heterogeneity of their parental cells, which makes them an important source of biomarkers (71). Specifically, their changes before and after treatment also show great potential in monitoring therapeutic response (72), thereby facilitating patient stratification and personalized cancer treatment. In particular, as a crucial medium for intercellular communication within the tumor microenvironment, EVs could be a key factor in monitoring the response to immunotherapy (73).

Depending on the cell that forms the EVs, they may contain active protein components, among which PD-L1 is also present. Therefore, detecting PD-L1 in EVs represents a viable approach. It has been confirmed that PD-L1 in EVs also contributes to immunosuppression, and its level is associated with the efficacy of ICIs (74). Christian Rolfo, a professor of medicine at the Icahn School of Medicine at Mount Sinai, and his team utilized PD-L1 in EVs extracted from blood to better predict and dynamically evaluate the response of non-small cell lung cancer(NSCLC) patients to cancer immunotherapy (75). EVs RNA expression in plasma has a statistically significant correlation with the efficacy of ICIs in melanoma and NSCLC (76). Studies have reported that in advanced NSCLC patients who did not respond to anti-PD-1 or anti-PD-L1 treatment, the levels of miRNA-200c-3p, miRNA-21-5p, and miRNA-28-5p in plasma EVs prior to treatment were elevated (20). Additionally, the combination of three biomarkers, miRNA-199a-3p, miRNA-21-5p, and miRNA-28-5p, was more effective in predicting immunotherapy response than PD-L1 expression detected by immunohistochemical assessment. MiRNAs such as miRNA - 320d, miRNA - 320c, and miRNA - 320b were able to predict partial responses of advanced NSCLC to ICIs (77).

However, currently, research in this area mainly focuses on malignant tumors such as lung cancer. The prediction and evaluation of EVs in the immunotherapy of HNSCC, especially neoadjuvant immunotherapy, remain under-explored. More studies are required to further elucidate its clinical value.

### 4.4 Methods based on nanotechnology

EVs possess several advantages over ctDNA and CTCs. As a result, this chapter will concentrate on the application of nanotechnology in the detection of EVs. The detection and molecular analysis of EVs present significant challenges, primarily due to their minuscule size and the difficulty in purifying them from multi-component serum or plasma samples.

Conventional methods for EVs separation, such as ultracentrifugation, ultrafiltration, or the use of kits, are known to be time-consuming, complex, and exhibit low separation efficiency. Similarly, conventional EVs analysis techniques, including polymerase chain reaction(PCR), Western blotting(WB), and enzyme-linked immunosorbent assay (ELISA), are not only time-consuming and labor-intensive but also have only moderate sensitivity. Moreover, these traditional methods often necessitate a separate EVs purification step, which further limits their practicality for clinical applications (78).

In recent times, nanomaterials, nanostructures, and nanotechnology have demonstrated remarkable advantages in the high-purity separation and high-sensitivity, high-specificity detection of EVs. Nanostructures and nanomaterials possess a large surface-to-volume ratio, which significantly increases the number of binding sites. This enhancement in binding sites directly leads to an improvement in the capture efficiency of EVs (79). Additionally, the nanoscale dimensions of these nanostructures enable the fabrication of substrates with densely packed nanostructures. Such substrates offer a unique opportunity to amplify the local signals emitted by the captured EVs (80). Nanomaterials and nanostructures employed for the separation and enrichment of EVs can generally be categorized into three distinct types: separation based on physical properties such as size, density, deformability, and charge; capture and isolation utilizing nanobeads; and enrichment facilitated by nanostructured substrates (80). Subsequently, the isolated EVs can be detected using various techniques, including fluorescence, surface plasmon resonance(SPR), surface-enhanced Raman spectroscopy (SERS), electrochemistry, and aptamers (78). Moreover, molecular biomarkers (such as proteins, DNA, and RNA) encapsulated within the EVs can be analyzed through immunostaining and sequencing methods (81). Simultaneously, nanometer characterization and analysis techniques, such as atomic force microscopy and nano-infrared spectroscopy, have the capability to detect single extracellular vesicles. This ability is highly beneficial for exploring the heterogeneity of extracellular vesicles and understanding its implications for tumor detection (82). The development of a plethora of novel technologies has substantially enhanced the detection efficiency of EVs and their associated cargoes.

However, despite these advancements, the widespread implementation of nanotechnology-based EVs detection in cancer management has not yet become a routine practice in clinical settings. The limited understanding of the correlation and efficiency of different nanotechnologies in EVs detection has posed a significant obstacle to the standardization and industrialization of these detection methods. Consequently, there is a pressing need for more in-depth clinical translational research in the future to bridge this gap and realize the full potential of nanotechnology in EVs detection for cancer diagnosis and treatment.

## 5 Radiology

Implementing immunotherapy within the therapeutic paradigm of HNSCC requires a comprehensive definition of therapeutic effectiveness. Historically, response assessment has been based on changes in tumor burden, both clinically and radiologically, according to the response evaluation criteria in solid tumors version 1.1 (RECIST v1.1) (83, 84). The immune-related response evaluation criteria in solid Tumors (iRECIST) is particularly suitable for this purpose because it has the same lesion selection and response evaluation criteria as RECIST v1.1 but requires confirmatory radiological follow-up at 4 to 8 weeks (85). However, all of these assessment methods are complex and often difficult to apply in clinical practice. RECIST v1.1 is still widely used, but its value is controversial (47, 85–87).

Radiomics is an auxiliary diagnostic technique for extracting large amounts of perfectly reproducible information from medical images. Radiomic analysis can effectively extract meaningful information from medical images, perform three - dimensional evaluations of tumors throughout the body, and conduct repeated evaluations during cancer treatment (88). Radiomics provides a valuable tool for the development of predictive biomarkers in the context of immunotherapy (89–92).

Radiomics has also been successfully used to predict PD-L1 expression in lung, esophageal, and urothelial cancers (93–95). Studies have constructed and validated radiomic signatures based on contrast-enhanced computed tomography to predict PD-L1 expression in HNSCC. Nine features were selected from the enhanced computed tomography of 157 patients with confirmed HNSCC to construct a radiomic feature model. The model showed good predictive efficacy and may help identify patients with HNSCC who could benefit from anti-PD-L1 immunotherapy (96). Immunotherapy has the potential to induce an early treatment response in certain patients with HNSCC. Such responses, however, cannot be diagnosed using conventional imaging parameters. Magnetic resonance imaging (MRI) diffusion-weighted imaging (DWI), on the other hand, may hold the key to detecting these early changes. One study was carried out to explore the correlation between early DWI parameters and the treatment response following immunotherapy for HNSCC. The researchers analyzed the imaging data of 24 patients with advanced squamous cell carcinoma both before and after immunotherapy. They found that round tumors with smaller diameters prior to treatment were more likely to exhibit a positive response. Additionally, a lower tumor skewness after treatment, along with a decreased overall skewness post-treatment compared to the pre-treatment state, was associated with a better treatment outcome (97). Another study established a multi-sequence MR volume histogram indicator model to predict the pathologic complete response (PCR) in patients with advanced HNSCC who were undergoing neoadjuvant chemotherapy immunotherapy (98). Furthermore, another research effort demonstrated the predictive value of multi-parameter MRI in evaluating the efficacy of neoadjuvant immunochemotherapy for locally advanced HNSCC (99).

In fact, the advent of radiomics for predicting immunotherapy responses has sparked a research frenzy in this field (100). Nevertheless, the absence of standardized protocols and validation procedures poses a significant challenge to its clinical application. Although numerous radiomics studies have attempted to predict responses across various tumor types, there remain inconsistencies in data selection, model construction, and outcome definition. These radiomics-based analyses still require validation in larger-scale clinical studies before they can be implemented in routine clinical practice.[18F] Fluorine deoxyglucose positron emission tomography/computed tomography (18F-FDG-PET/CT) has demonstrated its potential in the realm of immunotherapy. By utilizing metabolic response criteria, it can assess treatment responses and even provide prognostic information for patients, primarily those with non-small cell lung cancer and advanced melanoma (101). An 18F-FDG-PET/CT-based metabolic response assessment revealed that the calculated δ-value of focal total glycolysis (TLG) could be employed to detect pathological responses in HNSCC patients receiving neoadjuvant ICIs (102). While the accuracy at the primary tumor site reached 94%, this technique is limited by the relatively large tumor volume required to evaluate the influx of TLG and FDG from immune cells during ICI treatment, which may lead to false-positive results (103). In patients treated with ICIs, the resulting immune infiltration may further complicate the interpretation of PET/CT images. Shah et al. demonstrated that there was indeed no correlation between the changes in FDG uptake and the pathological tumor response following neoadjuvant PD-1 axis inhibition (104). To develop non-invasive methods for accurately assessing and predicting the tumor response to immunotherapy, a diverse range of affinity-based drugs targeting immune cell markers and checkpoint molecules have been developed and advanced to the clinical trial stage. Additionally, researchers have recently shifted their focus to substrate and activity - based imaging probes, which can offer real - time functional assessments of therapeutic immune responses. Besides the glucose metabolism of FDG, nucleic acid metabolism is also involved in the immunotherapy microenvironment (105). The changes in lipid metabolism of proliferating cells meet their increasing demands for energy and cell - membrane synthesis. [18F]-fluorocholine, a promising agent, can reflect the upregulation of cell-membrane synthesis (106, 107). Investigating whether the uptake of [18F]- fluorocholine or other lipid-metabolism-assessing imaging agents in lymphoid tissue can provide valuable information regarding the immune response to ICIs treatment would be an interesting research topic. The complexity of monitoring the tumor response in patients treated with ICIs has spurred the development of novel radiotracers. In particular, the PET/CT PD-L1 tracers currently used in clinical practice exhibit a strong correlation with PD-L1 status as measured by immunohistochemistry (108). Moreover, these tracers can display the heterogeneity of PD-L1 expression among different patients and within tumor lesions of the same patient on PET/CT, even more accurately than immunohistochemically stained biopsy samples (109). Our center participated in a study reporting a 68GA-labeled targeted covalent radiopharmaceutical fibroblast activator protein inhibitor (68Ga-TCR - FAPI), which demonstrated improved and sustained tumor targeting. It holds significant clinical value in medullary thyroid carcinoma, and its clinical translational value in evaluating the efficacy of tumor immunotherapy awaits further assessment (110). Compared with conventional radiology and FDG PET, radiotracers targeting immune cells pose additional challenges for image analysis. Given that CD8+ T cells play a crucial role in treatment response and clinical outcomes, the imaging agents being developed for non - invasive immune monitoring are designed to characterize different aspects of the CD8+ subpopulation. Infiltrating CD8+ cytotoxic T cells are unevenly distributed, with substantial differences within the same tumor lesion and between different tumor sites (111). Additionally, the dynamic nature of T- cell recruitment and activation makes it essential to optimize the imaging time after the commencement of treatment. Studying relevant quantitative indicators is also necessary. However, to our knowledge, only a few imaging probes are currently in the clinical research phase and have not yet received approval from the FDA for clinical use (112).

## 6 Artificial intelligence

Considering the complexity of the tumor microenvironment and the immune system, it is improbable that a single biomarker can be pinpointed to reliably describe prognosis and make predictions. Instead, an artificial intelligence (AI)-based approach holds the promise of defining new meta - biomarkers. This is achieved by integrating existing multi-omics datasets in oncology, which include genomics, pathology, radiomics, tumor microenvironment heterogeneity, and data generated from more real-world scenarios.

To develop more accurate prediction tools, machine learning algorithms have been utilized. These algorithms can exploit the nonlinear relationships between multiple variables, thereby achieving greater predictive power compared to a single biomarker. Currently, machine learning methods have been applied to analyze radiological signatures (91, 113), genetic signatures of the tumor microenvironment (114, 115), and hematoxylin and eosin images (116). Additionally, machine learning has been used to tentatively predict the immune - related adverse effects of immunotherapy (117, 118). However, previous studies have been restricted by small sample sizes and lack of validation, which has constrained their clinical application (119).

As a subset of artificial intelligence, machine learning (ML) encompasses a set of techniques. These techniques learn from data and iteratively enhance their performance to solve specific tasks, making use of available data about phenomena or processes. When the data consists of images, a standard ML model takes as input a set of predefined features (such as tumor shape, tumor size) that are extracted from the data, rather than the data in its original form. If an ample amount of data is accessible, deep learning (DL) can be employed. DL, a branch of ML, uses data in its raw format to discover and identify patterns, and it has been applied to tumor research (120, 121). Chowell et al. utilized a variety of clinical, genomic, and laboratory features to successfully develop a predictive model for the treatment response of ICIs across different cancers (122). Moreover, the random Forest ML tool has been validated for predicting the likelihood of ICIs response in patients with recurrent or metastatic head and neck squamous cell carcinoma (123).

The study affirms the growing utilization of artificial intelligence in uncovering predictive biomarkers of ICIs efficacy in diverse cancers. AI methods offer new perspectives from complex data. Nevertheless, the development of AI-based “software biomarkers” is impeded by retrospective datasets, inconsistent AI approaches, and unclear decision - making processes. Although these studies provide some insights that can generate hypotheses, their direct clinical implementation is limited. To create an explainable and accountable AI tool, large - scale prospective validation studies are essential (124).

## 7 Conclusion

The investigation of molecular markers for neoadjuvant immunotherapy in HNSCC represents a crucial breakthrough for individualized precision treatment. Current research has preliminarily validated the potential significance of various markers. These include the expression level of PD-L1, TMB, characteristics of the immune microenvironment (such as CD8+ T cell infiltration), gene expression profiles (like those of the IFN-γ signaling pathway), and specific gene variants in predicting the response to immunotherapy and patient prognosis. The development of non - invasive markers based on liquid biopsy, such as ctDNA, CTC, and EVs, offers novel directions for optimizing neoadjuvant immunotherapy strategies. Moreover, radiomics provides valuable insights into the exploration of molecular markers. Nevertheless, the existing markers are constrained by limitations in sensitivity and specificity. There is also a high degree of heterogeneity among different studies, along with a lack of comprehensive multi-omics analysis and prospective validation. In the future, it is imperative to construct multidimensional prediction models by integrating data from the genome, transcriptome, proteome, and spatial omics. Additionally, leveraging artificial intelligence technology to mine dynamic biomarkers is essential. The ultimate objective is to achieve the full-cycle management of “pre-treatment stratification, treatment-monitoring, and post-treatment evaluation” through the accurate identification of molecular markers. This will propel the immunotherapy of HNSCC from the realm of empirical medicine to a new era of evidence-based medicine.
